# Transmission dynamics and economic impacts of sheeppox and goatpox disease outbreak in Chifra district of Afar Region Ethiopia

**DOI:** 10.1016/j.heliyon.2022.e09674

**Published:** 2022-06-06

**Authors:** Belege Tadesse, Muhammed Hamid, Amin Hamid

**Affiliations:** a School of Veterinary Medicine, Wollo University, PO. Box. 1145, Dessie, Ethiopia; b College of Veterinary Medicine, Samara University, Afar, Ethiopia; c Chifra District Livestock Resource Office, Afar, Ethiopia

**Keywords:** Economic losses, Ethiopia, Goatpox, Sheeppox, Transmission dynamics

## Abstract

Sheep and goatpox are caused by pox virus and economically very important. The study was conducted to estimate the economic losses due to sheep and goatpox, to estimate the morbidity and mortality as well as the transmission parameters. A cross sectional study was conducted in Chifra districts of Afar region from July 2020 to December 2020 using questioner survey. For the estimation of the economic impacts and the transmission parameters of the outbreak, a data was collected at the end of the outbreak through a direct face to face interview. Transmission parameters were estimated based on a final size approach. Whereas, economic impacts were estimated descriptively using different formulas based on the type of losses. The overall morbidity, mortality and case fatality of sheep and goatpox were 51.6%, 2.0%, and 3.9%, respectively. The average flock level losses due to treatment cost, mortality and abortion were 320.3, 1250 and 1195.6 Ethiopian birr (ETB), respectively. The outbreak caused a total of 63617 ETB losses in the district. The highest loss was due to mortality (28750ETB), whereas the least loss was due to treatment cost (7367ETB). The outbreak had 0.14 and 1.41 transmission rate parameters per day and basic reproduction ratio, respectively. There was a significant difference in the transmission of the infection between individual animals (p < 0.001). To limit the economic losses due to this disease, the farmers should give more attention towards this disease and a systematic control program comprising vaccination and limitation of movement of sheep and goat should be implemented to alleviate the losses due to sheep and goatpox.

## 1 Introduction

Ethiopia has the largest livestock population in Africa with sheep and goat population exceeding 49 million [1]. Economically sheep and goats are the second most important animals next to cattle in the country [2]. Sheep and goats play a significant contribution to the domestic and export markets through the provision of non-food (manure, skin and wool) products, food (meat and milk) and also serves as a means of income by selling in the market [3].

Even if sheep and goat have a major role in the economy of the country, the benefit is constrained by several factors. From the constraints, livestock diseases are among the important ones that have hindered the development of the sector by decreasing production and hampering trade in animal and animal products [4]. Among these diseases, sheeppox and goatpox (SGP) are the most economically important diseases. It is caused by sheeppox and goatpox virus of the family Poxviridae, genus *Capripox virus.* In endemic areas, clinical sheeppox and goatpox are more commonly observed in exotic breeds as compared to indigenous breeds [5].

The disease is characterized by widespread skin eruption, generalized papules or nodules, fever, vesicles (rarely) on non-wool skin, paralysis, eruption in the form of red spots on the membranes of the eyes and nose, and on the wool-free parts of the skin. During post mortem examination lesions in the lung, respiratory and gastro-intestinal mucosa can also be observed [6]. It also leads to abortion in pregnant ewes and decreases in milk production. The fatality of the disease varies between different age groups of the animals. The disease is fatal in lambs. Whereas, it begins with high temperature and suppressed appetite in older sheep [7, 8].

Sheep and Goatpox viruses can survive for several weaks in nasal and oral secretion and for several months in scabs that have fallen off the animal. The virus can be transmitted during contact with infected animals and contaminated materials and by insects [9, 10].

Sheeppox and Goatpox are widely distributed in many countries including Asia and North and East Africa [11]. These diseases lead significant production loss because of decreased weight gain, increased abortion rates, damage to wool and hides, and increased susceptibility to pneumonia and fly strike, mortality [8, 12, 13]. Due to SGP outbreaks a loss of Indian rupee (INR) 107.5 million in Maharashtra, India [14], Rs. 2908.17, Rs. 4662.18 and Rs. 5599.06 in small, medium and large farms, respectively [13] and 81$ (13.7–792.6) in sheep and 117.4$ (9.8–609.5) [15] were reported.

Despite its considerable economic importance and threats to trade, information on the economic impact and transmission dynamics of the diseases in Ethiopia in general and in Afar region, in particular, is still scarce. Therefore, to design control measures the transmission parameters and impact of the SGP outbreak on the economy of the pastoralists should be investigated. Therefore, this study was conducted to estimate the morbidity and mortality rates, transmission parameters and economic impact of Sheep and Goatpox disease outbreak in Chifra District of Afar region.

## 2 Materials and methods

### 2.1 Study area

**Figure 1:**
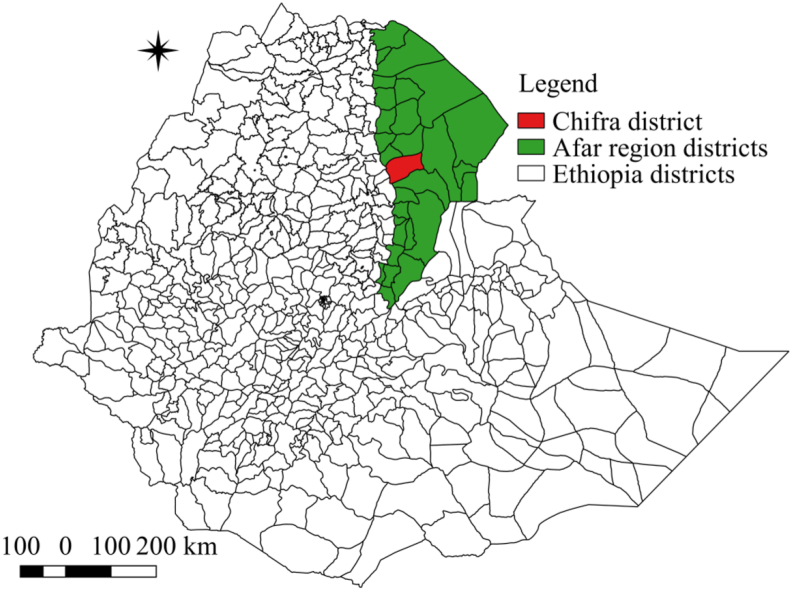
Map of the study area (Drawn by the authors using QGIS 2.18.28).

The study was conducted in Chifra district of Afar region from July 2020 to December 2020 (Figure 1). Chifra is located 162 km south west of Semera (Afar regional capital city). It has 173,374 ha of land in which the largest area is range land. It has an average temperature of 29 °C. The rainfall is bimodal with irregular distribution, with the long rainy season between Mid-June to Mid-September and the short rainy season between March and April. The average annual rainfall is recorded to be between 400 and 600 mm. Chifra is situated between >550–1,100 m above sea level [16]. In the district, sheep and goats are managed extensively and there is a mixing of different flocks in the field during grazing and watering.

### 2.2 Study design

A cross sectional investigation was used to study the transmission dynamics and the economic impact of Sheep and Goatpox disease outbreak in Chifra district of Afar region.

### 2.3 Study population

The study was conducted in a mixed population of sheep and goats that are managed in Chifra district affected by the Sheeppox and Goatpox outbreaks and were not vaccinated during the previous two years. All sheep and goat flocks that are considered as one epidemiological unit with the infected flocks (i.e. flocks having common grazing and watering) in the district being included in the study. Sheep and goats showing pox lesions like skin eruption, generalized papules or nodules, fever, vesicles (rarely) on non-wool skin, eruption in the form of red spots appearing on the membranes of the eyes and nose, and on the wool-free parts of the skin, were recognized as infected. The identification of infected sheep and goats were done by veterinarians in the district and researchers of this study.

### 2.4 Outbreak data collection

For both the transmission dynamics and the economic impact studies, the data was collected at the end of the outbreak using structured questioner (Annex) through a direct face to face interview of the owners. An outbreak is the occurrence of cases more than expected for shorter duration and covering a more limited area.

### 2.5 The basic reproduction ratio

The Basic reproduction ratio was estimated based on the final size of the outbreak [17]. It was estimated based on the total number of infected sheep and goats (shoat) at the end of the outbreak. Accordingly, it was estimated using the formula: –LN (1-p)/p; where LN is the natural logarithm and p is the proportion of infected shoats. The animals were considered infected when they show pox lesions clinically. Then from the basic reproduction ratio, the per day transmission rate parameter was estimated by considering a ten day infectious period for infected sheep and goat [18].

### 2.6 Morbidity, mortality and case fatality due to SGP outbreak

The morbidity and mortality due to the SGP outbreak were determined from the data collected at the end of the outbreak by a questioner survey. During the survey, farmers were asked to describe the clinical features of SGP that they observed on their animals during the outbreaks. If their description was approximately consistent with the key words used in the literature description of the disease, then for each category of animals, the number of animals at risk, affected, and dead due to SGP during the outbreak were recorded.

The animal level morbidity was determined as the proportion of animals infected during the outbreak and the herd level morbidity was determined as the proportion of herds infected. Mortality rate was determined as the proportion of died animals during the outbreak. Case fatality rate was estimated by dividing dead animals to the infected ones.

### 2.7 Economic impact assessment

The economic impact of SGP outbreak was estimated from the direct impact due to mortality loss, abortion and indirect impacts due to treatment cost [19].

The economic loss was estimated per outbreak, per flock and per animal. All the owners of a flock in the infected kebele were interviewed. The data on the economic losses were collected by interviewing each flock owner using a semi-structured questionnaire in the local language. Animal owners were interviewed about the number of sheep and goat infected, died, number of ewes and does that have abortion, cost of treatment for infected animals and time lost for the sake of treatment of infected animals.

#### 2.7.1 Mortality losses

The economic losses due to mortality per flock were estimated by considering the age categories (lamb, young and adult) and sex of animals that died and their corresponding market price using the following formula:

MSGPi = ∑ NMCij ∗ PCij.

where MSGPi represents the economic losses due to SGP induced death of flock i; NMCij is the number of animals that died in each category j of flock i and PCij is the price of that animal. The market price of each category of animals in each sex was judged by visiting the nearby markets and asking both animal owners and buyers; the average price for each category being used. Two markets in the nearby towns were visited to judge the price of animals. A total of 22 sellers and 17 buyers were asked and the average price was taken for each age category for both sexs. Information regarding the age of the animal was obtained from the owner of the animal and based on teeth structure of the animal and classified as lamb (birth to five month); young (5 months to ≤1.5 years) and adults (>1.5 years).

#### 2.7.2 Treatment cost

The economic cost of treatment was calculated using the following formula: TrCosti = (NTri∗PTri) + (NhoursLi∗Pdl), where TrCosti represents the treatment cost for affected flock i; NTri the number of animals treated in flocki; PTri the average per head expenditure to SGP treatment in flock i; NhoursLi the average number of working hours lost for seeking treatment for sick animals, and Pdl the average payment rate of a replacement laborer per hour in that locality. Treatment was given with antibiotics to avoid secondary bacterial complication.

#### 2.7.3 Abortion loss

Loss from abortion was estimated based on the number of abortion and the estimated price of lambs/kids in the study localities. All abortions in infected Ewe and Doe were recognized as due to sheep and goatpox [13]. The price of the lambs/kids was taken from their market price in the local market. Therefore, abortion loss was estimated by using the following formula:

ASGPi = ∑ NAi ∗ P;

where ASGPi is the economic losses due to SGP induced abortion of flock i; NAi is the number of ewes/doe encountered abortion in flock i and P is the average price of a lamb/kid.

Total economic loss due to the outbreak was estimated by adding the losses from death, abortion and treatment.

Losses due to reduction in the prices in animals affected were not considered because the aim of the owners of the affected flocks was not to sell animals in the short term.

#### 2.7.4 Data management and analysis

The collected data were edited and filtered using Microsoft Excel spreadsheet and analyzed by using STATA version 13. Descriptive statistics was used to estimate the economic impacts, morbidity and mortality rates. Transmission parameters were estimated based on a final size approach and a p-value of less than 0.05 was considered as statistically significant different.

## 3 Results

### 3.1 Description of the population

A total of 1485 sheep and goats (717 goats and 768 sheep) from 23 flocks that were considered as one epidemiological unit, were included in this study (Table 1).

**Table 1:** Structure of the study population.

Variable		Total	Sex			Age				Breed	
			Male		Female	Lamb	Young		Adult	Local	Exotic
**Species**	Sheep	768	192	576		75	214		479	768	0
	Goat	717	247	470		113	203		401	717	0
Overall		1485	439	1046		188	417	880		1485	0

### 3.2 Morbidity, mortality and case fatality rates of the outbreak

The overall morbidity, mortality and case fatality rates of the outbreak were 51.6, 2.0, and 3.9%, respectively (Table 2).

**Table 2:** Morbidity, Mortality and Case fatality rate of Sheep and goatpox disease outbreak at Chifra

Species	Total population	Number infected	Number dead	Morbidity rate (%)	Mortality rate (%)	Case fatality (%)
Goat	717	364	21	50.8	2.9	5.8
Sheep	768	402	9	52.3	11.7	2.2
Overall	1485	766	30	51.6	2.0	3.9

### 3.3 Transmission parameters of the outbreak

The per day transmission rate parameter and the basic reproduction ratio of the outbreak were 0.14 (0.10–0.26) and 1.41 (1.29–1.90) respectively. There was a significant difference in the transmission of the infection between individual animals (p < 0.001). The outbreak stays for 44 days from January 20/2020 to February 11/2020.

### 3.4 Economic impact of the outbreak

The outbreak results in a total of 63617 ETB losses in the district. The highest loss was due to mortality (28750ETB), whereas the least loss was due to treatment cost (7367ETB) (Table 3).

**Table 3:** Total economic losses due to the outbreak (in ETB).

	Sheep			Goat			Overall
	Lamb	Young	Adult	Kid	Young	Adult	
Treatment cost	-	-	-	-	-	-	7367
Mortality loss	8∗610 = 4880	4∗1300 = 5200	0	13∗720 = 9360	3∗1410 = 4230	2∗2540 = 5080	28750
Abortion loss	-	-	16∗500 = 8000	-	-	30∗650 = 19500	27500
Total	4880	5200	8000	9360	4230	24580	63617

ETB = Ethiopian Birr.

36 ETB = 1 USD during the study period.

The average flock level losses due to treatment cost, mortality and abortion, related to the outbreak were 320.3, 1250 and 1195.6 ETB, respectively (Table 4).

**Table 4:** Flock level average economic losses (in ETB).

	Sheep			Goat			Overall
	Lamb	Young	Adult	Kid	Young	Adult	
Treatment cost							320.3
Mortality loss	212.2	226.1	-	407	183.9	220.9	1250
Abortion loss	-	-	347.8	-	-	847.8	1195.6
Total	212.2	226.1	347.8	407	183.9	1068.7	2766

## 4 Discussion

The current study estimates the morbidity, mortality, case fatality, transmission parameters and economic impact of SGP disease outbreak in Chifra districts of Afar region based on the current outbreak that happened in the district. The overall morbidity, mortality and case fatality rates of the current outbreak were 51.6, 2.0, and 3.9% respectively. These findings are lower than the morbidity of 75–100% and case fatality of between 10 and 85% depending on the virulence of the virus [20]. It is also lower than the report of Garner *et al.* [12], who reported an average morbidity and mortality rates of 63.50% and 49.50%, respectively, in Maharashtra state of India. The morbidity rate is higher than the report of Venkatesan et al. [21] and Soundararajan et al. [22], who reported a morbidity rate of 11.4%, and 43% respectively. Whereas, the case fatality is lower than the 60% case fatality of outbreak in India- Maharashtra in 2007 as reported by Venkatesan et al. [21]. The current finding agrees with the report of AHA [23] who reported morbidity rates ranging from 1% to 75% or higher and a mortality rate of less than 10% in indigenous breeds of goats. Variations in morbidity, mortality and case fatality would be due to variation in the breed of the animal, its immunity to SGP virus, the strain of the virus and the variation of weather condition. Mild infections are common among indigenous breeds in endemic areas, but more severe disease can be seen in young or stressed animals and those with concurrent infections, or from areas free of pox disease previously.

Based on species, morbidity, mortality and case fatality were 50.8 %. 2.9 % and 5.8 % for goat and 52.3 %, 11.3 % and 2.7 % in sheep in the current study. Abdi [24] reported a morbidity, mortality and case fatality rates of 32.1%, 4.7% and 14.5%, respectively in sheep and 29.4%, 6.5% and 22%, respectively in goat in Adea Berga district, West Shoa zone, Central Ethiopia [24].

The current outbreak in Chifra district of Afar region results in a total of 63617 ETB losses in the district. The highest loss was due to mortality (28750 ETB), whereas the least loss was due to treatment cost (7367 ETB). This loss agrees with the report of Senthilkumar and Thirunavukkarasu [13] who reported that the loss due to mortality of affected animals is the highest, ranging from 40 to 60% of total loss in different farm size categories in Tamil Nadu in India.

The average flock level losses due to treatment cost, mortality and abortion of the current were 320.3, 1250 and 1195.6 respectively. A median flock level economic loss of 81$ (13.7–792.6) in sheep and 117.4$ (9.8–609.5) in goat were reported by Limon et al. [15] from Northeast Nigeria. Senthilkumar and Thirunavukkarasu [13] also reported an average annual loss due to sheeppox was found to be Rs. 2908.17, Rs. 4662.18 and Rs. 5599.06 in small, medium and large farms, respectively. According to Senthilkumar and Thirunavukkarasu [13], the overall per animal economic loss due to sheeppox in ram, ewe and lamb was Rs.1048.81, Rs. 744.26 and Rs. 200.22, respectively. Garner et al. [14] also reported losses due to SGP outbreaks in Maharashtra, INR 107.5 million was incurred from 5000 infected flocks.

The current outbreak has 0.14 (0.10–0.26) and 1.41 (1.29–1.90) per day transmission rate parameter and basic reproduction ratio, respectively. These findings indicate the occurrence of an outbreak and the basic reproduction ratio of the outbreak indicates that on average one infected animal transmits the virus to more than one animal during its infectious period. But the number of animals that can be infected by different infectious animal varies significantly. This variation could be due to the probability of getting contact of each infected animals with other susceptible animal and the probability of transmission during each contact. There was no other previous study that determines the transmission parameter and basic reproduction ratio for SGP disease, making comparisons of the current finding is very difficult. The basic reproduction number was slightly low; this may be because of the fact that during the outbreak the contact rate between infected and susceptible animals is limited. Molla et al. [18] estimated a basic reproduction ratio of 1.21 (95% CI: 1.01–1.46) for outbreaks of LSD in Ethiopia, which is one of the poxvirus disease that affects mainly cattle's. Based on the basic reproduction number from the current study, to achieve a herd immunity vaccinating 47.4% of the population is mandatory.

## 5 Conclusion

Sheep and goatpox disease is one of the contagious and economically important disease. A high level of morbidity and relatively low mortality and case fatality rates were occurred during the current outbreak. The severe economic losses estimated to be arising from the sheeppox and goatpox outbreak indicate that this disease remains as a serious economic problem. Relatively moderate transmission parameters were estimated for the outbreak. Since the disease causes high morbidity rate and economic losses, the farmers should give more attention towards this disease through creation of adequate awareness among them through suitable extension programmes for prevention and control of the disease. A systematic control program comprising vaccination and limitation of movement should be implemented to alleviate the losses due to sheeppox and goatpox disease.

## Declarations

### Author contribution statement

Belege Tadesse: Analyzed and interpreted the data; Contributed reagents, materials, analysis tools or data; Wrote the paper.

Muhammed Hamid: Conceived and designed the experiments; Analyzed and interpreted the data.

Amin Hamid: Conceived and designed the experiments; Performed the experiments.

### Funding statement

This research did not receive any specific grant from funding agencies in the public, commercial, or not-for-profit sectors.

### Data availability statement

Data will be made available on request.

### Declaration of interest’s statement

The authors declare no conflict of interest.

### Additional information

No additional information is available for this paper.

## Figures and Tables

**Table:** 

District	Kebele	Herd owner	Date	Species	Age category	No. of new cases	No. ​of infectious animals	No. ​of susceptible animals	No. ​of recovered ​animals	Total animals per flock
										
										

**Table:** 

Flock no.	Owner name	Total shoat per herd		Total infected		Total died			No. of ewe &Doe aborted		Treatment cost per herd	Time spent to take infected shoats to clinic	Remark
		sheep	goat	Sheep	Goat	Lamb & kid (up to 5 month	Young (up to 2 yrs	Adult (>2 yrs)	Sheep	goat			
1													
2													
3													
4													
5													
6													
7													
8													
9													
10													
11													
12													
13													
14													
15													
16													
17													
18													
19													
20													
21													
22													
23													
24													
25													

## Annex

Questionnaire format for interviewing smallholder farmers in Chifra district to collect data on SGP outbreak occurred in the area.

### II. Morbidity and Mortality Data recording format for Sheep and goats

1. Do you have sheep and goats? Yes No.

2. If yes, did the SGP affect your sheep? Yes No.

3. How many of your sheep are affected? (Affected/total number).

3.1. For lambs (pre-weaning) _________/__________

3.2. For yearling_________/___________

3.3. For adult___________/__________

4. How many of your sheep and goat died? (Died/total number/unit price).

4.1. For lambs (pre-weaning) _________/__________/________

4.2. For yearling_________/___________/_________________

4.3. For adult___________/__________/__________________

4.4. Do you slaughter infected animals? Yes No.

4.5. If you slaughter infected shoat for what purpose do you use the skin?

 Selled _____ for home use _____ other______

4.6. If the skin is rejected from the market what is the average price of the skin in the local market?_________________________________

5. How many ewes/does abort?_______________________________

6. Do you treat year infected sheep and goat? Yes No.

6.1. If you say yes how much do you pay per sheep?________________________

7. Data recording format for Sheep and goatpox outbreak at Chifra District, Afar region, 2020.
